# Antigen-Presenting Cells Represent Targets for R5 HIV-1 Infection in the First Trimester Pregnancy Uterine Mucosa

**DOI:** 10.1371/journal.pone.0005971

**Published:** 2009-06-22

**Authors:** Romain Marlin, Marie-Thérèse Nugeyre, Claire de Truchis, Nadia Berkane, Amélie Gervaise, Françoise Barré-Sinoussi, Elisabeth Menu

**Affiliations:** 1 Institut Pasteur, Regulation of Retroviral Infection Unit, Department of Virology, Paris, France; 2 Gynecology-Obstetrics Service, A. Béclère Hospital, AP-HP, Clamart, France; 3 Department of Gynecology Obstetrics and Reproductive Medecine, Tenon Hospital, AP-HP, UMPC, Paris, France; Comprehensive AIDS Reseach Center, China

## Abstract

**Background:**

During the first trimester of pregnancy, HIV-1 mother-to-child transmission is relatively rare despite the permissivity of placental cells to cell-to-cell HIV-1 infection. The placenta interacts directly with maternal uterine cells (decidual cells) but the physiological role of the decidua in the control of HIV-1 transmission and whether decidua could be a source of infected cells is unknown.

**Methodology/Principal Findings:**

To answer to this question, decidual mononuclear cells were exposed to HIV-1 *in vitro*. Decidual cells were shown to be more susceptible to infection by an R5 HIV-1, as compared to an X4 HIV-1. Infected cells were identified by flow cytometry analysis. The results showed that CD14^+^ cells were the main targets of HIV-1 infection in the decidua. These infected CD14^+^ cells expressed DC-SIGN, CD11b, CD11c, the Fc gamma receptor CD16, CD32 and CD64, classical MHC class-I and class-II and maturation and activation molecules CD83, CD80 and CD86. The permissivity of decidual tissue was also evaluated by histoculture. Decidual tissue was not infected by X4 HIV-1 but was permissive to R5 HIV-1. Different profiles of infection were observed depending on tissue localization.

**Conclusions/Significance:**

The presence of HIV-1 target cells in the decidua *in vitro* and the low rate of *in utero* mother-to-child transmission during the first trimester of pregnancy suggest that a natural control occurs *in vivo* limiting cell-to-cell infection of the placenta and consequently infection of the fetus.

## Introduction

During pregnancy, mother-to-child transmission (MTCT) of HIV-1 is relatively rare. Indeed, most MTCT cases occur during delivery and in the *postpartum* period via breastfeeding [1], [2]. Even in the absence of antiretroviral therapy, 90% of fetuses are protected against *in utero* transmission. Most *in utero* transmissions occur during the third trimester of pregnancy [2]. This suggests that a natural control of MTCT occurs particularly during the first months of pregnancy. In the uterine cavity the placenta acts as a physical barrier between the fetus and the mucosa of the uterine wall (also known as the decidua) [3]. We and others have shown that the placenta plays a role in controlling HIV-1 transmission, since the trophoblast cells, which form the outer layer of the placenta, are not permissive to cell-free HIV-1 virus [4]–[6]. However trophoblast cells can be infected via cell-to-cell contact by HIV-1 infected cells [7], [8]. Cytokines (such as TNFα and IL-8 among others), and chemokines (such as CCL5 and LIF) present in the placental environment regulate this route of infection [8], [9].

Primary contact of trophoblast cells during pregnancy is mainly with decidual cells during the first trimester and with maternal blood cells thereafter [10]. Therefore the rarity of MTCT during the first trimester might be explained either by the absence of HIV-1 target cells in the decidua, or, if target cells are present, by a natural control of viral transmission occurring within the decidual tissue.

In the first trimester of pregnancy, decidual tissue is defined by localization and function: the decidua basalis is localized at the implantation site in close contact with the placenta, the decidua parietalis lines the rest of the uterine wall, and the decidua capsularis surrounds the conceptus [11]. The immune cells of the decidua are composed of a high number of natural killer (dNK) cells (which have a different phenotype and functions than their blood counterparts), antigen presenting cells (dAPCs), some regulatory T lymphocytes and a very small percentage of other innate immunity cells such as NKT cells or γδ T lymphocytes [12]. From the beginning of pregnancy these immune cells regulate the balance between inflammatory events required for placental invasion and angiogenesis [13], [14] and anti-inflammatory reactions needed for fetus survival [15]. These events are dependent on cell-to-cell interactions between immune cells and trophoblast cells.

The main objectives of this study were to investigate the permissivity of the decidual cells to cell-free HIV-1 and to identify potential HIV-1 target cells. Since MTCT events are rare during the first trimester of pregnancy, the presence of target cells within the decidua would strongly suggest that a first step of control occurs *in vivo* to limit the dissemination of the virus from infected decidual cells to the trophoblast. We focused our study mainly on the decidua basalis due to the direct contact of this tissue with the trophoblast.

## Results

### The decidual CD14^+^ cells constitute potential targets of HIV-1 infection

**Figure 1:**
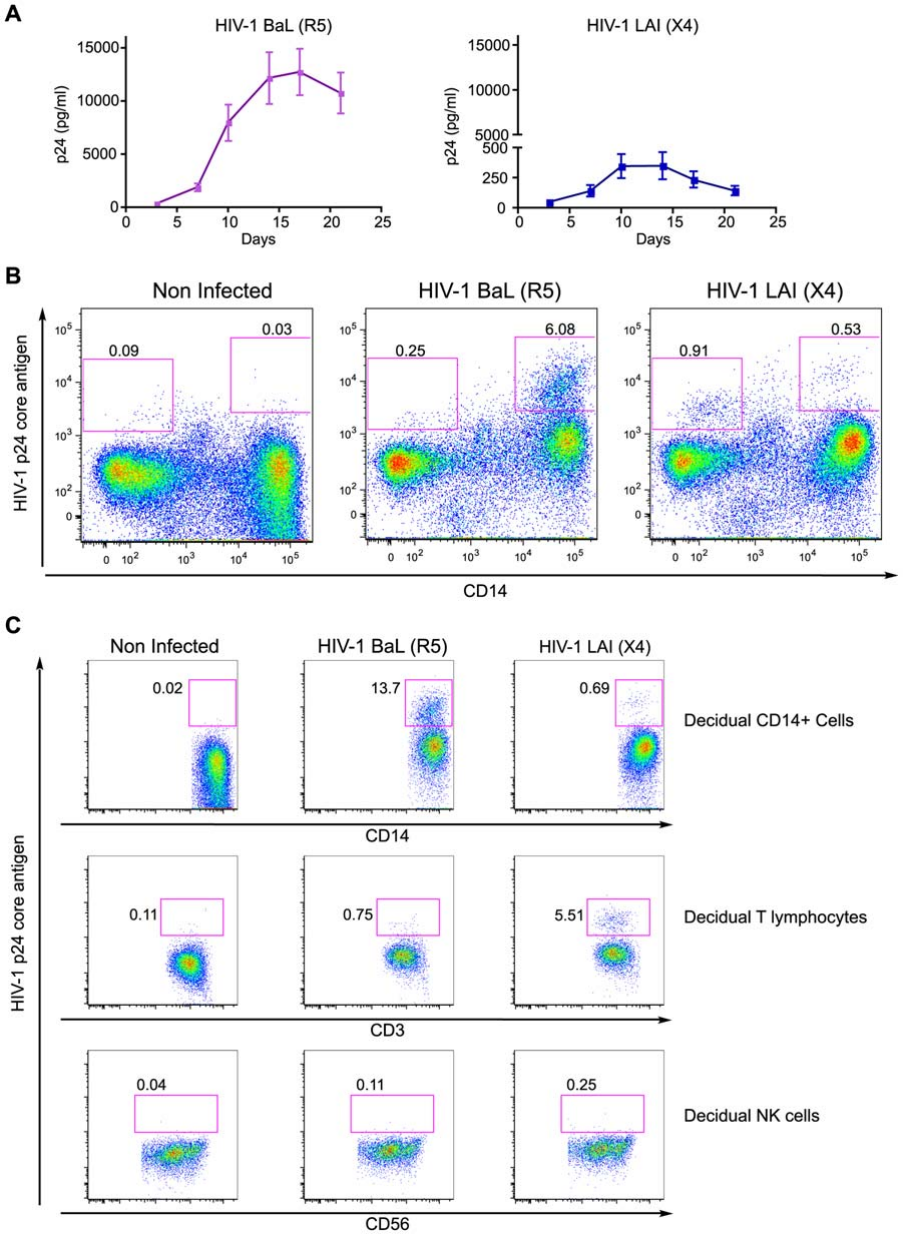
Flow cytometry analyses of decidual infected cells. Isolated mononuclear cells of decidua basalis were infected with HIV-1_BaL_ (R5) and HIV-1_LAI_ (X4) at 10^−3^ MOI. (A) Results are expressed in pg/ml of HIV-1 p24 viral core antigen measured by ELISA during the time course experiment and represents the mean of p24 antigen detection in supernatant from twelve different donors. (B) Decidual cells were analyzed at 11 days post infection. Cells were gated on the leukocyte population (CD45^+^) and characterized by expression of CD14 and p24 antigen markers. (C) To evaluate the level of infection in each type of leukocytes, cells were also gated on CD14^+^ cells (top), CD3^+^ cells (middle) and CD56^+^ cells (bottom). Each pink gate shows the p24 antigen positive cells. Pink gates also determine the background for non-infected condition. Flow cytometry pictures (B and C) show a representative of nine different donors.

To identify potential cellular targets of HIV-1, mononuclear cells were isolated from decidua basalis tissue and infected with HIV-1_BaL_ (R5) or HIV-1_LAI_ (X4). Decidual leukocyte population was composed by dNK cells (mean 64.48%, +/−8.81 SD), CD14^+^ dAPC (mean 20.92%, +/−6.29 SD), CD4^+^/CD3^+^T lymphocytes (mean 4.74%, +/−1.82 SD) and non-CD4 CD3^+^ T lymphocytes (mean 6.64%, +/−2.99 SD) (n = 12). Viral infection was monitored by the amount of the viral core p24 antigen in supernatant over time (Fig. 1A). Viral replication of the HIV-1_BaL_ strain in decidual mononuclear cells was higher than in the HIV-1_LAI_ infection. The mean p24 antigen production in HIV-1_LAI_ infected cell supernatant was more than 35 fold lower than the one observed in HIV-1_BaL_ infected cell supernatant. Flow cytometry analyses of these cells were performed at day 11 post-infection when viral production was still increasing *in vitro*. Infected cells were identified with an anti-p24 antigen antibody in the leukocyte population (CD45^+^ cells) and were characterized with anti-CD14, anti-CD3 and anti-CD56 monoclonal antibodies. Flow cytometry analysis showed that CD14^+^ cells are the main targets of HIV-1 infection in this system (6,09%) (Fig. 1B). CD14^+^ cells were infected with HIV-1_BaL_ (13,7%) and poorly infected by HIV-1_LAI_ (0,69%) (Fig. 1C). CD3^+^ lymphocytes were poorly infected with HIV-1_BaL_ (0,75%), infection by HIV-1_LAI_ was higher (5,51%) (Fig. 1C). However HIV-1_LAI_ infected CD3^+^ cells constituted a small percentage of the leukocytes population (0,76%) compared to HIV-1_BaL_ infected CD14^+^ cells (6,09%) (data not shown). Flow cytometry analyses also showed that no detectable intracellular p24 was found in CD56^+^ NK cells neither with HIV-1_BaL_ nor with HIV-1_LAI_ (Fig. 1C). These data are representative of analysis from nine different donors. Since the highest percentage of p24^+^ cells is detected in HIV-1_BaL_ infected CD14^+^ cells, we can conclude that the CD14^+^ cells are the main target of HIV-1_BaL_ infection. Moreover, decidual cells are more susceptible to HIV-1_BaL_ infection than HIV-1_LAI_ as shown by the frequency of p24^+^ cells.

**Figure 2:**
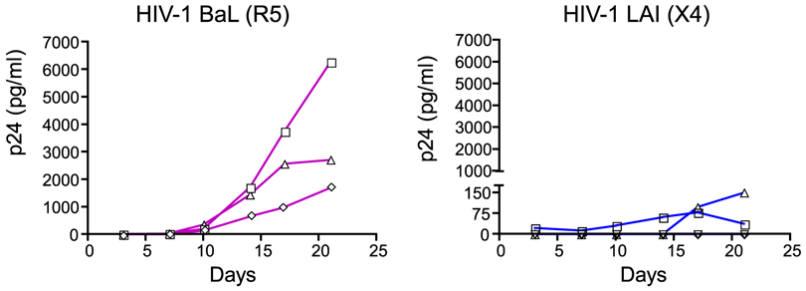
Infection of purified decidual CD14^+^ cells. Purified decidual CD14^+^ cells were infected by HIV-1_BaL_ (R5) and HIV-1_LAI_ (X4) strains (10^−3^ MOI), and cultured up to 21 days. Infections were monitored by p24 antigen quantification over the course of the experiment. Each line represents a separate donor. CD14^+^ cells were purified from deciduas from at least three different donors.

To determine whether the isolated decidual CD14^+^ cells could be permissive to HIV-1, they were purified from HIV-1 negative decidual tissue with magnetic beads and subsequently infected. As shown in Figure 2, decidual CD14^+^ cells were susceptible to infection by HIV-1_BaL_ (ranging from 1700 to 6280 pg p24 Ag/ml), but were not permissive to HIV-1_LAI_ (max = 150 pg p24 Ag/ml). However, the amount of p24 antigen detected in decidual CD14^+^ cells culture supernatant infected with HIV-1_BaL_ was approximately 35 fold lower than the amount detected in culture supernatant of peripheral CD14^+^ macrophages (obtained as previously described [16]) (data not shown). These results indicate that decidual CD14^+^ cells were permissive to HIV-1_BaL_ but to a lower extent than peripheral blood macrophages.

### Phenotypic characterization of HIV-1 infected decidual CD14^+^ cells

**Figure 3:**
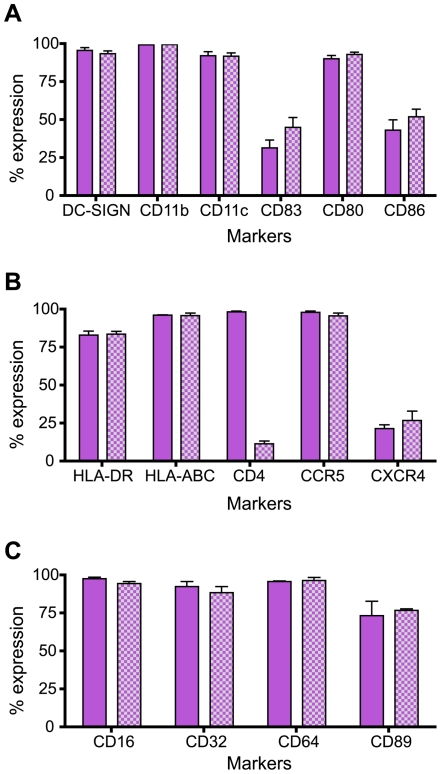
Phenotypic characterization of infected decidual CD14^+^ cells. Isolated mononuclear cells from decidua basalis were infected with HIV-1_BaL_ (R5) at 10^−3^ MOI. Cells were analyzed 11 days post infection. Cells were gated on leukocyte population (CD45^+^) and CD14^+^ cells in non-infected cells or CD14^+^/p24^+^ cells in infected cells. Results are represented in percentage of expression in CD14^+^ cells (purple bars) or CD14^+^/p24^+^ infected cells (checkered bars). Analyses of decidual cells from nine different donors are represented with standard error of the mean as error bars. (A) Expression of DC-SIGN, myeloid markers CD11b and CD11c, maturation marker CD83 and activation markers CD80 and CD86 were evaluated. (B) MHC-II and classical MHC-I molecules, CD4 HIV-1 receptor and HIV-1 co-receptors of entry expression were also measured. (C) Expression of Fc receptors.

To characterize the phenotype of the infected decidual CD14^+^ cells, flow cytometry analyses were performed on infected decidual mononuclear cells at day 11 post infection. We observed that infected decidual CD14^+^ are CD11b^+^, CD11c^+^ and DC-SIGN^+^ (Fig. 3A). They also expressed the co-stimulation markers CD80 and CD86, and the maturation marker CD83 at a lower level. They expressed high levels of Fc gamma receptors CD16 (FCγRIII), CD32 (FCγRII) and CD64 (FCγRI); and the Fc alpha receptor CD89 (Fig. 3C). As expected, down regulation of CD4 was observed on infected cell surface but HLA-DR and HLA-ABC remained highly expressed. Infected CD14^+^ cells all expressed CCR5, the R5 HIV-1 co-receptor and at a lower level CXCR4, the X4 HIV-1 co-receptor (Fig. 3B). These results indicate that infected decidual CD14^+^ cells express cell surface molecule that are used as markers to define dendritic cells and macrophages.

### Decidual histocultures are permissive to R5 HIV-1_BaL_

**Figure 4:**
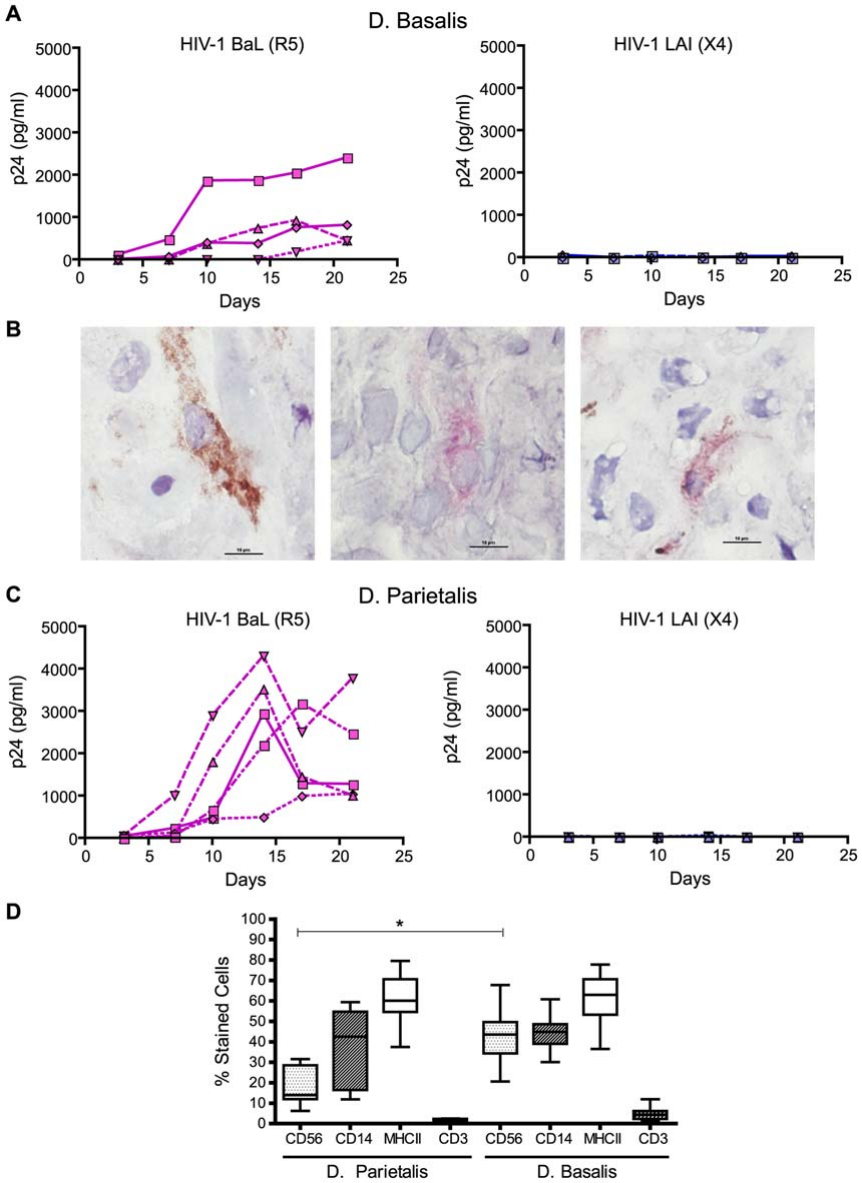
Infection of decidua basalis and decidua parietalis histocultures. (A) Histocultures of decidua basalis were infected with HIV-1_BaL_ (R5) (left) and HIV-1_LAI_ (X4) (right) virus at 1,800 TCID_50_/explant and cultured for 21 days following extensive washes. Each line represents a separate donor. Results are expressed in pg/ml of p24 antigen measured by ELISA. At least three different deciduas were tested for each series of infection. (B) Infected cells were identified and phenotyped on a decidua basalis section at day 10 post infection by HIV-1_BaL_. Frozen sections were stained with anti-CD14 (left) (brown cells) and anti-p24 antigen (middle) (red cells). Infected cells were characterized by co-expression of the two markers (right). Images were taken at ×600 magnification and black scale line represents 10 µm length. (C) Histocultures of decidua parietalis were infected with HIV-1_BaL_ (left) and HIV-1_LAI_ (right) HIV-1 with the same conditions of histocultures of decidua basalis. (D) Proportion of the main leukocytes population was evaluated by immunostaining in decidua parietalis and decidua basalis histocultures at day 0. CD56^+^, CD14^+^, MHC-II^+^ and CD3^+^ cells were quantified on tissue sections from at least 3 different deciduas. Minimums of 500 cells were evaluated on each decidua.

To evaluate the permissivity of the decidual tissue to HIV-1 infection, the histoculture model [17] was adapted to the decidua basalis (DB). The morphology of the tissue sections at different time points showed that decidual tissue does not display major structural alterations before day 18 (Fig. S1). Viral infection was monitored by the amount of the viral core p24 antigen in supernatant over time (Fig. 4A). No consistent productive HIV-1 infection was observed in DB tissue when HIV-1_LAI_ was used for infection. Moreover no productive infection was observed even with a 10 fold higher viral exposure (data not shown). Conversely, DB tissue was infected by HIV-1_BaL_ with production level of p24 antigen ranging from 500 to 2000 pg/ml.

To confirm the phenotype of the infected cells *in situ* in DB histocultures, double immunohistochemistry staining was performed on infected tissue sections. As shown in Figure 4B, infected cells co-expressing the CD14 surface marker and the intracellular p24 antigen were found in the decidual tissue.

As decidual immune populations are known to be different depending on the localization of the tissue, we also evaluated the permissivity of decidua parietalis (DP) tissue to HIV-1 (Fig. 4C). As was observed for DB tissue, no productive infection was detected with HIV-1_LAI_ in DP histoculture supernatant. This tissue was permissive to infection by HIV-1_BaL_, but different infection profiles were observed when DP and DB infected histocultures were compared. We observed a higher production level of p24 antigen in DP histocultures with a maximum viral production around day 14.

Since different profiles of infection were shown between the tissues, both DB and DP histocultures were analyzed by immunohistochemistry at Day 0 (Fig. 4D) to evaluate the percentage of the main leukocyte populations in the tissue. As expected, high numbers of decidual NK cells (dNK) (CD56^+^ cells), and decidual antigen presenting cells (CD14^+^ cells) were present in both decidual tissue explants. A higher proportion of dNK was found in the DB (median 43.53%, +/−12.68 SD) than in the DP (median 13.95%, +/−9.3 SD) (p = 0.001). CD14^+^ cells also constituted a major population in both tissue types (median in DB 44.85%, +/−7.29 SD and median in DP 42.54%, +/−17.93 SD). A high proportion of HLA class II–expressing cells (median DP 60.13% and DB 62.93%) and a low number of CD3^+^ cells (median DP 1.52% and DB 4.44%) were also observed in histocultures. These experiments demonstrate that DB and DP contain the same proportion of CD14^+^ cells while differences in infection profile were observed.

Taken together, these results showed that decidua could be permissive to HIV-1_BaL_ both at cellular and tissue level and that the decidual CD14^+^ cells were the main HIV-1_BaL_ target *in vitro* and *in situ* in histoculture.

## Discussion

Several studies have shown the roles of immune cell populations of the decidua (human uterine mucosa during pregnancy) in early pregnancy events. Indeed, fetus tolerance involves dAPCs [18], [19] and decidual T regulatory lymphocytes [20], the dNK cells are crucial for angiogenesis [21], [22] and trophoblast invasion [13], [23]. These immune cells also have an important role in the fetus implantation [23]. In contrast, their role in the control of viral transmission remains to be defined, particularly question of the permissivity of these mucosal cells to HIV-1 infection. Here we report for the first time that decidual mucosa and decidual mononuclear cells are permissive to HIV-1 *ex vivo*. We observed consistent infection with an HIV-1_BaL_ strain (R5) but poor infection with an HIV-1_LAI_ strain (X4), demonstrating that these cells were permissive mainly to R5 virus.

Flow cytometry analyses showed that T lymphocytes are permissive to HIV-1_LAI_. However this population represents a small proportion of decidual leukocytes and HIV-1_LAI_ infection is poor or undetectable in the mononuclear cells or at the tissue level when compared to HIV-1_BaL_ infection. The sensitivity of ELISA assays and the influence of the cytokine environment could also explain the detection of infected HIV-1_LAI_ CD14^+^ cells by flow cytometry but the lack of detectable p24 production by HIV-1_LAI_ infected purified decidual CD14^+^ cells and histocultures.

We identified that the main target cells for R5 HIV-1 in the decidua are CD14^+^ cells. In decidual tissue, the CD14 molecule is expressed by the antigen presenting cell populations, which are mostly macrophages and dendritic cells [24]. Phenotypic characterization of decidual dendritic cells remains a topic of debate. In some reports, dendritic cells are defined by the absence of lineage antigens including the CD14 molecule [25]; in other reports these cells express the CD14 receptor [19]. Here, we report that the main HIV-1 target cells bear markers of both DC and macrophages, which are usually used to distinguish these types of cells in the peripheral blood. According to a recent study [26], the decidual CD14^+^ cells that we characterize as CD11b^+^, CD11c^+^, DC-SIGN^+^, CD16^+^, CD32^+^, CD64^+^, HLA-DR^+^, HLA-ABC^+^, CD4^+^, CD80^+^, CD86^+/−^ and CD83^+/−^ cells could be Alternatively Activated M2 Macrophages [27]. These types of macrophages are known to possess immunoregulative and tissue remodeling functions [27] that are crucial at the materno-fetal interface [28]. Cells with this same phenotype are also found in the thymus but are described as dendritic cells [29]. These thymic DC are permissive to HIV-1 R5 but not to HIV-1 X4 infection [30].

We also show that purified decidual CD14^+^ cells replicate the R5 virus HIV-1_BaL_, but to a lower level than peripheral blood macrophages. This difference of replication level could be due to the nature of decidual CD14^+^ cells. A recent study has shown that the polarization of M1 and M2a macrophages is associated with a reduced capacity to support productive R5 HIV-1 infection [31]. As M2 macrophages are heterogenous [27] and HIV-1 infected decidual CD14^+^ cells bear macrophage and DC markers, further investigation of the function and the phenotype of infected decidual CD14^+^ cells is required.

The presence of HIV-1 target cells in the decidua indicates that a natural control might occur *in vivo* since mother-to-child transmission events are rare during the first trimester of pregnancy. Decidual CD14^+^ cells, which are the main decidual targets of HIV-1 R5 infection, are present in similar numbers in the decidua basalis (DB) and in the decidua parietalis (DP). This suggests that the number of target cells for HIV-1 infection is comparable between DP and DB. However we observed different profiles of productive viral infection between the two tissue types of decidua, and a higher production of virus was observed in DP infection compared to DB infection. As the number of target cells is comparable in both tissues, the different infection profiles suggest that there may be a limitation of HIV-1 R5 infection in the decidua basalis. The lack of detection of HIV-1 X4 productive infection could also be due to a control at the decidua level. Even if the placenta has been shown to be an efficient barrier against HIV-1 transmission by several non-exclusive mechanisms, a first level of control might occur in the decidua to limit the dissemination of the virus that could be transmitted by cell-to-cell contact from infected decidual cells to the trophoblast cells of the placenta [4]. Several mechanisms of control may be involved; including maternal innate immunity. Scott-Algara et al observed an activating phenotype of peripheral blood NK cells in HIV-1 exposed but uninfected patients [32], suggesting a protective role of activated NK cells in HIV-1 infection. Other studies recently showed a control of HIV-1 replication in dendritic cells mediated by peripheral blood NK cells [33]. The activation phenotype of dNK cells [22], [34] suggests a role for these cells in the control of HIV-1 infection within the decidua. Furthermore, at the materno-fetal interface, a subtle balance of pro- and anti-inflammatory cytokine secretion is required for the invasion of the placenta and the maintenance of pregnancy [15]. We, and others [8], [9], have previously shown that some of these cytokines can inhibit (LIF, CCL-5) or enhance (TNF-α, IL-8) the passage of the virus through the placental barrier. They might also play a role in the control of viral dissemination within the decidua.

The role of decidual immune cells in the control of mother-to-child transmission of pathogens is relatively uncharacterized. Previous reports on this subject describe interactions between decidual immune cells (dAPCs) and Cytomegalovirus [35] or between *Listeria monocytogenes* and dNK cells [36] or dAPCs [37]. Here, for the first time, we demonstrate the susceptibility of decidual mucosa to HIV-1 infection in isolated mononuclear cells as well as at the tissue level. The identification of HIV-1 decidual target cells, the description of different permissivity to X4 and R5 virus, and the fact that the risk of mother-to-child transmission is very low during the first trimester of pregnancy underline that the materno-fetal interface is a very relevant model to study the mechanism of natural protection against HIV-1 transmission at a mucosal level.

## Materials and Methods

### Ethics statement

All women provided their written informed consent. The study was approved by the ethics committee of the Hôtel Dieu (Paris, France), the Assistance Publique des Hôpitaux de Paris (n° VAL/2006/06-41/01) and the biomedical research committee of the Institut Pasteur, Paris, France (n° RBM/2005.024).

### Human decidual tissue collection

Decidual tissue samples were obtained from healthy women undergoing voluntary pregnancy termination at the first trimester of pregnancy (6–10 weeks) at Antoine Béclère Hospital, Clamart, France and Tenon Hospital, Paris, France.

Decidua parietalis and decidua basalis were isolated from the other tissues collected and washed with RPMI 1640 Glutamax medium (Gibco, Invitrogen, Cergy Pontoise, France) supplemented with 10% of foetal calf serum to eliminate blood and placental contaminants.

### HIV-1 isolates

Infections were performed with HIV-1 prototype isolates: BaL (R5) which use CCR5 as entry co-receptor and LAI (X4) which use CXCR4 as entry co-receptor. Viral isolates were amplified on PHA-stimulated PBMC from two blood donors for 10 days. PBMC cultures were maintained in RPMI 1640 Glutamax (Gibco) supplemented with 10% of foetal calf serum, penicillin, streptomycin and 100 U/ml of recombinant Interleukin-2 (Chiron, Netherlands). Viral isolates were concentrated by centrifugation on Vivaspin 100,000 Kda columns (Sartorius, Palaiseau, France) at 1400×g for 40 minutes. The tropism of viral isolates was confirmed by infection of U87-CD4, U87-CD4/CCR5 and U87-CD4/CXCR4 cells as previously described [38].

### Antibodies

The antibodies used for flow cytometry and immunohistochemistry experiments are listed in Table 1.

**Table 1:** 

Abs anti-	Species/clone	Manufacturer	use
CD14 purified	Mouse/RMO52	Beckman Coulter	IHC*
CD14-Pacific Blue	Mouse/M5E2	BD	FLC*
CD3-PE-Texas Red	Mouse/UCHT1	Beckman Coulter	FLC
CD45-Amcyan	Mouse/2D1	BD	FLC
CD56-Alexa fluor 700	Mouse/B159	BD	FLC
DC-SIGN-PE	Mouse/120507	R&D	FLC
CD11b-APC-Cy7	Mouse/ICRF44	BD	FLC
CD11c-PE-Cy7	Mouse/3.9	eBioscience	FLC
CD80-PE	Mouse/L307.4	BD	FLC
CD83-APC	Mouse/HB15e	BD	FLC
CD86-APC	Mouse/2331	BD	FLC
CD16-PE-Cy5	Mouse/3G8	Beckman Coulter	FLC
CD32-APC	Mouse/FL18.26	BD	FLC
CD64-PE-Cy5	Mouse/22	Beckman Coulter	FLC
CD89-PE	Mouse/A59	BD	FLC
HLA-DR-PE	Mouse/Immu357	Beckman Coulter	FLC
HLA-ABC-PE-Cy5	Mouse/W6/32	eBioscience	FLC
CD4-PE-Cy7	Mouse/SK3	BD	FLC
CXCR4-APC	Mouse/12G5	BD	FLC
CCR5-APC-Cy7	Mouse/2D7	BD	FLC
p24-FITC	Mouse/KC57	Beckman Coulter	FLC
CD3 purified	Mouse/F7.2.38	Dako	IHC
CD56 purified	Mouse/N901	Beckman Coulter	IHC
MHC-II purified	Mouse/CR3/43	Dako	IHC
p24 (HIV-1 core antigen)	Goat/polyclonal	Abcam	IHC
Biotin labelled donkey anti-goat	Donkey/polyclonal	Abcam	IHC
HRP polymer labelled	Goat anti-mouse	Dako	IHC
Isotype matched Ig controls	Mouse	Beckman Coulter	FLC, IHC

* FLC: Flow cytometry, IHC: Immunohistochemistry.

### Decidual histocultures

Decidual tissues (parietalis or basalis) were cut in 0.3 cm^2^ pieces and placed on collagen sponge gels (Pfizer, Paris, France) in 1.5 ml/well/sponge/piece of rich culture media containing Ham F12: DMEM glutamax (V∶V) (Gibco) supplemented with 15% of foetal calf serum (PAA, Les Mureaux, France), penicillin (0.1 U/l) and streptomycin (1×10^−8^ g/l) (Gibco). Histocultures were cultured for 21 days.

### Isolation of decidual mononuclear cells

Freshly isolated decidual basalis tissue was cut into small fragments and digested for 1 hour at 37°C under agitation in PBS (Gibco) with 1 mg/ml collagenase IV (Sigma, St Quentin Fallavier, France) and 50 U/ml recombinant DNase I (Roche, Meylan, France). The cell suspension was filtered through 100, 70 and 40 µm sterile nylon cell strainer successively (BD Biosciences, Le pont de Claix, France). The mononuclear cell population was isolated using Lymphocyte Separation Medium (PAA) and cultured in the rich culture media Ham F12: DMEM glutamax. Positive selection of CD14^+^ cells was performed using anti-CD14 magnetic beads (Miltenyi, Paris, France) as recommended by the manufacturer.

### HIV-1 infection of decidual histocultures

Explants of decidua parietalis and decidua basalis were incubated overnight with the different HIV-1 isolates or mock infected PBMC supernatants, at 1,800 TCID50 (optimized viral dose) per explant except where indicated. Explants were then washed 3 times and placed on collagen sponge gels for 21 days in 1.5 ml culture medium/well/sponge/piece. Culture supernatants were harvested and fresh medium added to each well every 3–4 days. HIV-1 p24 antigen was quantified in the culture supernatants by ELISA (Zeptometrix, Gentaur). Experiments were performed in triplicate and for each virus, decidua from at least 3 different donors were tested.

In some experiments, decidual tissues were also collected at different time points and snap-frozen in Tissue-Tek for immunohistochemistry.

### HIV-1 infection of isolated decidual mononuclear cells and of purified CD14 positive cells

Isolated decidual mononuclear cells were incubated 2 hours with HIV-1 isolates at 10^−3^ MOI or mock supernatant. Cells were washed 3 times and cultured at 10^6^ cells/ml. Cells were analysed by flow cytometry at different time points. Infected cells were stained for surface markers and subsequently fixed with 1% paraformaldehyde solution (EMS, Hatfield, USA). For intracellular staining of p24 antigen, cells were permeabilized using IntraPrep reagent (Beckman Coulter) according to manufacturer’s instructions. The antibodies used for the experiments are shown in Table 1. Flow cytometry analysis was performed using LSRII 2-Blue 2-Violet 3-Red 5-Yelgr lasers configuration (BD Biosciences), using Diva (BD Biosciences) and FlowJo 8.8.5 (Tristar) software. At least 1000 events in p24^+^ gate were analysed in each test.

### Immunohistochemistry

Decidual sections were obtained by embedding decidual tissue (at different culture time points) in Tissue-Tek (Sakura, Gentaur, Paris, France) and snap-frozen in an isopentane/liquid nitrogen bath. Frozen Tissue-Tek blocks were cut with a cryostat and frozen sections (5 µm thick) were fixed in acetone and rehydrated in TBS (Dako, Trappes, France). To evaluate the immune cell population and identify infected cells, tissue sections were double stained for HIV-1 core p24 antigen (goat antibody) and different surface markers (mouse antibody) described in Table 1. Endogenous peroxidase and alkaline phophatase (AP) were blocked for 10 minutes by addition of hydrogen peroxide and levamisol (Dako). Surface markers were visualized using the Envision+ dual link system (Dako) and Histogreen (Abcys, Paris, France) as a peroxidase substrate. HIV-1 core antigen was visualized using biotin-streptavidin-alkaline phosphatase complex and Vector Red (Abcys), as an AP substrate. Tissue sections were counterstained with haematoxylin (Labonord, templars, France), mounted in permanent medium and analysed with a Nikon microscope Eclipse 80i.

### Statistical analysis

All experiments were performed in at least three independent assays. A Wilcoxon test was used for comparison of immune cell subpopulations in both types of decidua. A p-value of <0.05 was considered to be significant.

## Supporting Information

Figure S1 Tissue sections of decidua basalis histocultures at different culture time points. Structure and morphology of histoculture explants was evaluated at day 0 (A), 3 (B), 6 (C), 10 (D), 14 (E), 18 (F) and 21 (G). Cell nuclei were stained with haematoxylin (blue) and blood vessel structure was visualized on tissue sections (black arrows). Necrotic zones appeared from day 18 (grey arrows). Pictures were taken at ×100 magnification and the black scale-line represents 100 µm length.
(9.36 MB TIF)
