# Supplementary figures and images for: Antigen-Presenting Cells Represent Targets for R5 HIV-1 Infection in the First Trimester Pregnancy Uterine Mucosa
Source: PLoS One. 2009 Jun 22;4(6):e5971. doi: 10.1371/journal.pone.0005971 (PMC2696085; doi:10.1371/journal.pone.0005971)

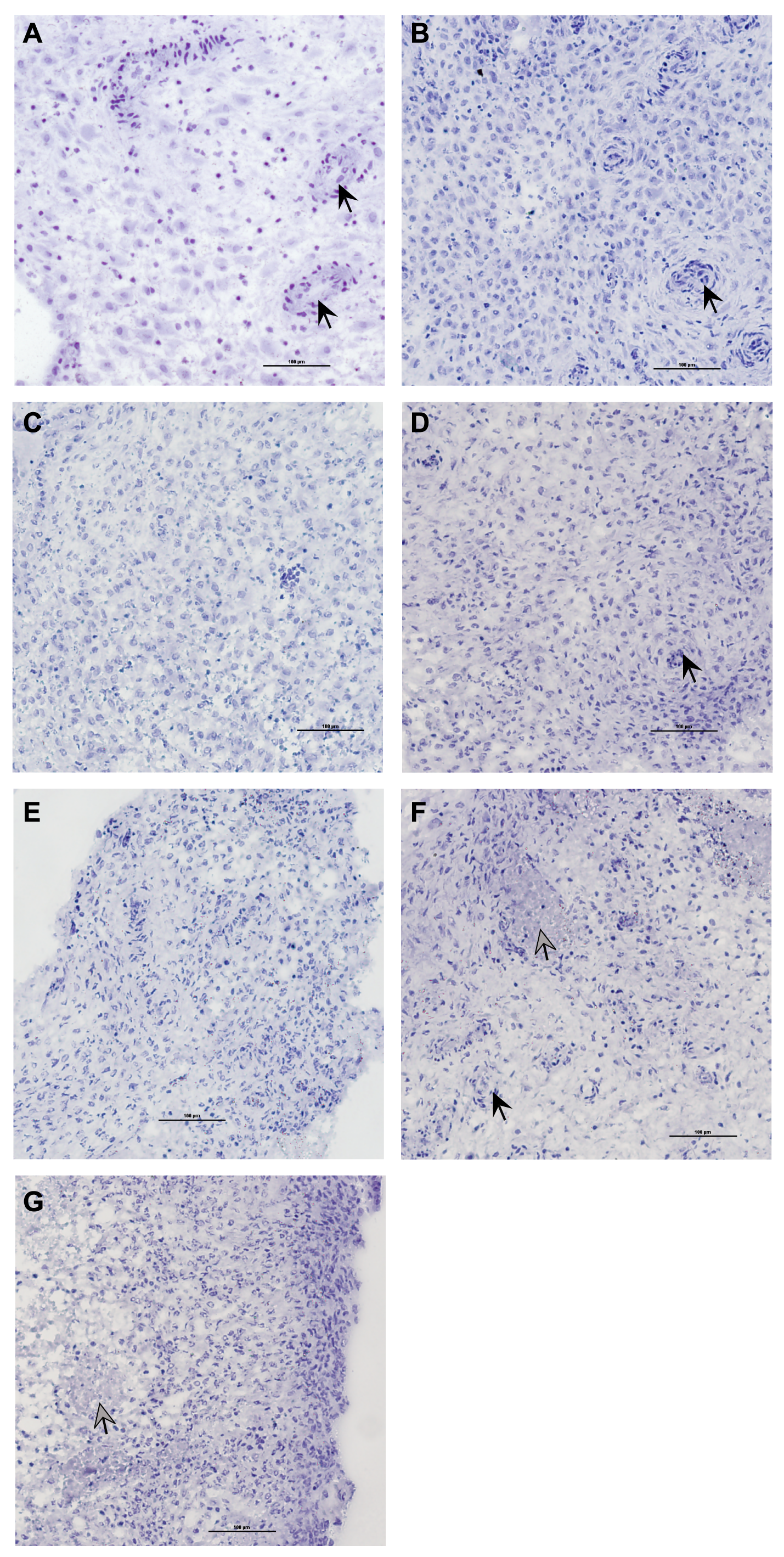

Supplement: Figure S1 — Tissue sections of decidua basalis histocultures at different culture time points. Structure and morphology of histoculture explants was evaluated at day 0 (A), 3 (B), 6 (C), 10 (D), 14 (E), 18 (F) and 21 (G). Cell nuclei were stained with haematoxylin (blue) and blood vessel structure was visualized on tissue sections (black arrows). Necrotic zones appeared from day 18 (grey arrows). Pictures were taken at ×100 magnification and the black scale-line represents 100 µm length. (9.36 MB TIF) [file pone.0005971.s001.tif]
